# Chloridobis{*N*-[(dimethyl­amino)­dimethyl­sil­yl]-2,6-dimethyl­anilido-κ^2^ *N*,*N*′}titanium(III)

**DOI:** 10.1107/S1600536810025092

**Published:** 2010-07-03

**Authors:** Juan Chen

**Affiliations:** a Department of Chemistry, Taiyuan Teachers College, Taiyuan 030031, People’s Republic of China

## Abstract

In the monomeric title titanium(III) compound, [Ti(C_12_H_21_N_2_Si)_2_Cl], the metal atom is surrounded by two *N*–silylated anilide ligands in an *N*,*N*′′-chelating mode. The two ends of the N—Si—N chelating unit exhibit different affinity to the metal center. The Ti—N_amine_ bond is longer than the Ti—N_anilide_ bond by about 0.29 Å. The two ligands are arranged *trans* to each other and the mol­ecule demonstrates a pseudo-twofold rotation along the axis of the Ti—Cl bond. The five–coordinate Ti atom demonstrates a highly distorted trigonal-bipyramidal geometry.

## Related literature

For related titanium compounds, see: Ovchinnikov *et al.* (1993 ▶); Chomitz *et al.* (2008 ▶). For amido titanium compounds as olefin polymerization catalyts, see: Alesso *et al.* (2008 ▶); Oakes *et al.* (2004 ▶); Tabernero *et al.* (2009 ▶). For catalytic applications of related *N*–silylated analido group-4-metal compounds towards olefin polymerization, see: Gibson *et al.* (1998 ▶); Hill & Hitchcock (2002 ▶); Yuan *et al.* (2010 ▶). For related organometallic compounds with analogous analido ligands, see: Schumann *et al.* (2000 ▶); Chen (2008 ▶, 2009 ▶).
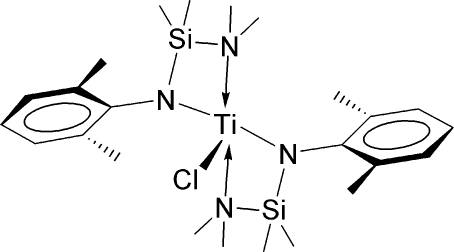


## Experimental

### 

#### Crystal data

[Ti(C_12_H_21_N_2_Si)_2_Cl]
*M* *_r_* = 526.12
Monoclinic, 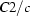

*a* = 34.145 (5) Å
*b* = 9.2718 (15) Å
*c* = 20.909 (3) Å
β = 122.894 (2)°
*V* = 5558.2 (15) Å^3^
*Z* = 8
Mo *K*α radiation
μ = 0.51 mm^−1^
*T* = 213 K
0.40 × 0.30 × 0.15 mm

#### Data collection

Bruker SMART area-detector diffractometer
Absorption correction: multi-scan (*SADABS*; Sheldrick, 1996 ▶) *T*_min_ = 0.814, *T*_max_ = 0.928
10981 measured reflections
4866 independent reflections
4473 reflections with *I* > 2σ(*I*)
*R*_int_ = 0.033

#### Refinement

*R*[*F* ^2^ > 2σ(*F* ^2^)] = 0.072
*wR*(*F* ^2^) = 0.168
*S* = 1.17
4866 reflections
289 parameters
H-atom parameters constrained
Δρ_max_ = 0.47 e Å^−3^
Δρ_min_ = −0.48 e Å^−3^

### 

Data collection: *SMART* (Bruker, 2000 ▶); cell refinement: *SAINT* (Bruker, 2000 ▶); data reduction: *SAINT*; program(s) used to solve structure: *SHELXS97* (Sheldrick, 2008 ▶); program(s) used to refine structure: *SHELXL97* (Sheldrick, 2008 ▶); molecular graphics: *SHELXTL* (Sheldrick, 2008 ▶); software used to prepare material for publication: *SHELXL97*.

## Supplementary Material

Crystal structure: contains datablocks I, global. DOI: 10.1107/S1600536810025092/rk2217sup1.cif

Structure factors: contains datablocks I. DOI: 10.1107/S1600536810025092/rk2217Isup2.hkl

Additional supplementary materials:  crystallographic information; 3D view; checkCIF report

## Figures and Tables

**Table 1:** Selected bond lengths (Å)

Ti1—N1	1.989 (3)
Ti1—N3	1.995 (3)
Ti1—N2	2.282 (4)
Ti1—N4	2.291 (4)
Ti1—Cl1	2.3374 (13)

**Fig. 1.**
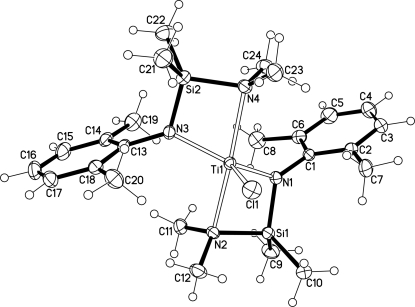
The molecular structure, showing the atom–numbering scheme. Displacement ellipsoids are drawn at the 30% probability level. H atoms are presented as a small spheres of arbitrary radius.

## supplementary crystallographic information

### Comment

Group 4 metal amides supported with the *N*–silylated anilido ligands were active catalysts for olefin polymerization (Gibson *et al.*, 1998; Hill & Hitchcock, 2002). In particular, titanium amides were found to be more efficient and applicable (Alesso *et al.*, 2008; Oakes *et al.*, 2004; Tabernero *et al.*, 2009). Therefore, the monoionic *N*–silylated anilido ligand bearing a pendant amino group was employed for synthesizing titanium compound. Analogous compounds with different metals including Zn (Schumann *et al.*, 2000), Zr (Chen, 2009) and Fe (Chen, 2008) have been synthesized. Moreover, a group of zirconium amides with the similar ligand were reported showing good performance in ethylene polymerization (Yuan *et al.*, 2010). It implied that the title titanium compound would behave better in catalysis application.

The title compound was prepared by the metathetical reaction of TiCl_4_(*THF*)_2_ with [LiN(Si*Me*_2_N*Me*_2_)(2,6-*Me*_2_C_6_H_3_)]_2_. It is interesting that the valence of Ti has changed from IV to III. Similar situation could be found in Ovchinnikov's work (Ovchinnikov *et al.*, 1993) and other - Chomitz *et al.*, 2008). The driving factors for reduction will be investigated in further research. The suitable single–crystal of the title compound was obtained by recrystallization in toluene. Its molecular structure is shown in Fig. 1. In the monomeric molecular structure of title compound, the metal center is coordinated by two *N*–silylated anilido ligands. Each ligand has an N—Si—N chelating moiety, which is presumed to be a "quasi" conjugated unit owing to *d*···π–interaction between Si and N atoms. Two ligands are arranged in *trans*– to each other and obey the *pseudo–C_2_* symmetrical operation. Such arrangement makes Ti atom right in the triangular planes of N1···N3···Cl1 and N2···N4···Cl1. The five–coordinate Ti(III) center demonstrates a highly distorted trigonal–bipyramid geometry (N2– and N4–apical atoms). The configuration is as same as the Fe(III) compound reported previously (Chen, 2008), presumably due to the same valence. The metal center Ti is chelated with an average N—Ti—N bite angle of 74.18 (13)°. The corresponding N—Si—N of the ligand is constrained to be about 95.25 (16)°. The mean Ti—N_anilido_ bond is 1.992 (3)Å, whereas the mean Ti—N_amino_ bond is 2.286 (4)Å in the  title compound. It suggests the former is much tighter than the latter. They are different from corresponding bond lengths 1.972 (4)Å and 2.356 (6)Å in a related amido Ti(III) compound reported by Chomitz *et al.* (2008).

### Experimental

TiCl_4_(*THF*)_2_ (0.48 g, 1.45 mmol) was added into the solution of [LiN(Si*Me*_2_N*Me*_2_)(2,6-*Me*_2_C_6_H_3_)]_2_ (0.66 g, 1.45 mmol) in *Et*_2_O (30 ml) at 273 K. The reaction mixture was warmed to room temperature and kept stirring for 12 h. It was dried in vacuum to remove all volatiles and the residue was extracted with CH_2_Cl_2_ (30 ml). Concentration of the filtrate under reduced pressure and recrystallization in toluene gave the title compound as purple crystals (yield 0.50 g, 66%).

### Refinement

The methyl H atoms were constrained to an ideal geometry, with C—H distances of 0.97Å and *U*_iso_(H) = 1.5*U*_eq_(C), but each group was allowed to rotate freely about its C—C, C—N and C—Si bonds. The other H atoms were placed in geometrically idealized positions and constrained to ride on their parent atoms, with C—H distances in the range 0.94Å and *U*_iso_(H) = 1.2*U*_eq_(C).

### Crystal data

**Table:**

[Ti(C_12_H_21_N_2_Si)_2_Cl]	*F*(000) = 2248
*M**_r_* = 526.12	*D*_x_ = 1.258 Mg m^−^^3^
Monoclinic, *C*2/*c*	Mo *K*α radiation, λ = 0.71073 Å
Hall symbol: -C 2yc	Cell parameters from 4223 reflections
*a* = 34.145 (5) Å	θ = 2.3–27.6°
*b* = 9.2718 (15) Å	µ = 0.51 mm^−^^1^
*c* = 20.909 (3) Å	*T* = 213 K
β = 122.894 (2)°	Block, purple
*V* = 5558.2 (15) Å^3^	0.40 × 0.30 × 0.15 mm
*Z* = 8	

### Data collection

**Table:**

Bruker SMART area-detector diffractometer	4866 independent reflections
Radiation source: fine–focus sealed tube	4473 reflections with *I* > 2σ(*I*)
graphite	*R*_int_ = 0.033
φ and ω scans	θ_max_ = 25.0°, θ_min_ = 1.4°
Absorption correction: multi-scan (*SADABS*; Sheldrick, 1996)	*h* = −40→40
*T*_min_ = 0.814, *T*_max_ = 0.928	*k* = −6→11
10981 measured reflections	*l* = −24→24

### Refinement

**Table:**

Refinement on *F*^2^	Primary atom site location: structure-invariant direct methods
Least-squares matrix: full	Secondary atom site location: difference Fourier map
*R*[*F*^2^ > 2σ(*F*^2^)] = 0.072	Hydrogen site location: inferred from neighbouring sites
*wR*(*F*^2^) = 0.168	H-atom parameters constrained
*S* = 1.17	*w* = 1/[σ^2^(*F*_o_^2^) + (0.062*P*)^2^ + 21.4919*P*] where *P* = (*F*_o_^2^ + 2*F*_c_^2^)/3
4866 reflections	(Δ/σ)_max_ = 0.001
289 parameters	Δρ_max_ = 0.47 e Å^−^^3^
0 restraints	Δρ_min_ = −0.48 e Å^−^^3^

### Special details

**Table:**

Geometry. All s.u.'s (except the s.u. in the dihedral angle between two l.s. planes) are estimated using the full covariance matrix. The cell s.u.'s are taken into account individually in the estimation of s.u.'s in distances, angles and torsion angles; correlations between s.u.'s in cell parameters are only used when they are defined by crystal symmetry. An approximate (isotropic) treatment of cell s.u.'s is used for estimating s.u.'s involving l.s. planes.
Refinement. Refinement of *F*^2^ against ALL reflections. The weighted *R*–factor *wR* and goodness of fit *S* are based on *F*^2^, conventional *R*–factors *R* are based on *F*, with *F* set to zero for negative *F*^2^. The threshold expression of *F*^2^ > σ(*F*^2^) is used only for calculating *R*–factors(gt) *etc*. and is not relevant to the choice of reflections for refinement. *R*–factors based on *F*^2^ are statistically about twice as large as those based on *F*, and *R*–factors based on ALL data will be even larger.

### Fractional atomic coordinates and isotropic or equivalent isotropic displacement parameters (Å^2^)

**Table:**

	*x*	*y*	*z*	*U*_iso_*/*U*_eq_	
Ti1	0.15222 (2)	0.34275 (8)	0.06414 (4)	0.0231 (2)	
Si1	0.14374 (4)	0.45890 (13)	0.18120 (7)	0.0284 (3)	
Si2	0.12637 (4)	0.23530 (14)	−0.08132 (7)	0.0291 (3)	
Cl1	0.23284 (4)	0.30582 (13)	0.13779 (7)	0.0399 (3)	
N1	0.12378 (11)	0.3088 (4)	0.12435 (18)	0.0249 (7)	
N2	0.15461 (11)	0.5585 (4)	0.11873 (19)	0.0278 (8)	
N3	0.12846 (11)	0.4008 (4)	−0.04285 (18)	0.0266 (8)	
N4	0.13576 (12)	0.1310 (4)	−0.0022 (2)	0.0302 (8)	
C1	0.09894 (14)	0.1945 (4)	0.1318 (2)	0.0255 (9)	
C2	0.12258 (15)	0.0776 (5)	0.1805 (2)	0.0310 (10)	
C3	0.0969 (2)	−0.0339 (5)	0.1844 (3)	0.0461 (13)	
H3A	0.1126	−0.1127	0.2166	0.055*	
C4	0.0490 (2)	−0.0317 (6)	0.1420 (3)	0.0547 (15)	
H4A	0.0321	−0.1075	0.1460	0.066*	
C5	0.02597 (18)	0.0816 (6)	0.0941 (3)	0.0492 (14)	
H5A	−0.0068	0.0823	0.0649	0.059*	
C6	0.04990 (15)	0.1948 (5)	0.0877 (3)	0.0347 (10)	
C7	0.17487 (16)	0.0709 (5)	0.2274 (3)	0.0430 (12)	
H7A	0.1849	−0.0171	0.2572	0.064*	
H7B	0.1871	0.1536	0.2612	0.064*	
H7C	0.1864	0.0719	0.1940	0.064*	
C8	0.02325 (15)	0.3130 (6)	0.0313 (3)	0.0456 (13)	
H8A	−0.0099	0.2956	0.0064	0.068*	
H8B	0.0314	0.3148	−0.0065	0.068*	
H8C	0.0311	0.4050	0.0576	0.068*	
C9	0.10009 (19)	0.5429 (6)	0.1969 (3)	0.0472 (13)	
H9A	0.1148	0.5659	0.2504	0.071*	
H9B	0.0746	0.4760	0.1814	0.071*	
H9C	0.0882	0.6305	0.1669	0.071*	
C10	0.19967 (18)	0.4424 (6)	0.2747 (3)	0.0453 (12)	
H10A	0.1962	0.4827	0.3142	0.068*	
H10B	0.2238	0.4945	0.2734	0.068*	
H10C	0.2082	0.3415	0.2856	0.068*	
C11	0.11430 (17)	0.6473 (5)	0.0631 (3)	0.0384 (11)	
H11A	0.1152	0.7391	0.0861	0.058*	
H11B	0.0856	0.5973	0.0481	0.058*	
H11C	0.1157	0.6639	0.0186	0.058*	
C12	0.19724 (17)	0.6475 (5)	0.1534 (3)	0.0409 (11)	
H12A	0.1927	0.7351	0.1740	0.061*	
H12B	0.2038	0.6722	0.1150	0.061*	
H12C	0.2233	0.5937	0.1941	0.061*	
C13	0.12235 (15)	0.5317 (5)	−0.0824 (2)	0.0293 (10)	
C14	0.07746 (16)	0.5910 (5)	−0.1291 (2)	0.0354 (11)	
C15	0.0718 (2)	0.7169 (6)	−0.1689 (3)	0.0481 (13)	
H15A	0.0418	0.7561	−0.2004	0.058*	
C16	0.1094 (2)	0.7862 (6)	−0.1633 (3)	0.0550 (15)	
H16A	0.1050	0.8702	−0.1916	0.066*	
C17	0.1533 (2)	0.7303 (5)	−0.1156 (3)	0.0440 (12)	
H17A	0.1790	0.7789	−0.1104	0.053*	
C18	0.16069 (17)	0.6047 (5)	−0.0751 (3)	0.0357 (11)	
C19	0.03519 (16)	0.5235 (6)	−0.1355 (3)	0.0473 (13)	
H19A	0.0077	0.5798	−0.1702	0.071*	
H19B	0.0395	0.5215	−0.0857	0.071*	
H19C	0.0313	0.4258	−0.1547	0.071*	
C20	0.20968 (17)	0.5505 (6)	−0.0233 (3)	0.0490 (13)	
H20A	0.2311	0.6145	−0.0262	0.074*	
H20B	0.2122	0.4543	−0.0388	0.074*	
H20C	0.2173	0.5478	0.0287	0.074*	
C21	0.17307 (18)	0.1982 (6)	−0.0987 (3)	0.0476 (13)	
H21A	0.1593	0.1624	−0.1502	0.071*	
H21B	0.1942	0.1264	−0.0628	0.071*	
H21C	0.1901	0.2864	−0.0924	0.071*	
C22	0.06979 (18)	0.1932 (6)	−0.1711 (3)	0.0499 (13)	
H22A	0.0756	0.1580	−0.2089	0.075*	
H22B	0.0509	0.2799	−0.1900	0.075*	
H22C	0.0535	0.1200	−0.1612	0.075*	
C23	0.17362 (19)	0.0209 (6)	0.0299 (3)	0.0484 (13)	
H23A	0.1629	−0.0638	−0.0026	0.073*	
H23B	0.1819	−0.0059	0.0806	0.073*	
H23C	0.2007	0.0606	0.0325	0.073*	
C24	0.09293 (18)	0.0616 (6)	−0.0162 (3)	0.0447 (13)	
H24A	0.0875	−0.0273	−0.0443	0.067*	
H24B	0.0667	0.1260	−0.0455	0.067*	
H24C	0.0965	0.0403	0.0321	0.067*	

### Atomic displacement parameters (Å^2^)

**Table:**

	*U*^11^	*U*^22^	*U*^33^	*U*^12^	*U*^13^	*U*^23^
Ti1	0.0230 (4)	0.0245 (4)	0.0225 (4)	−0.0011 (3)	0.0128 (3)	0.0006 (3)
Si1	0.0335 (6)	0.0254 (6)	0.0285 (6)	−0.0036 (5)	0.0184 (5)	−0.0021 (5)
Si2	0.0284 (6)	0.0325 (7)	0.0267 (6)	−0.0002 (5)	0.0153 (5)	−0.0005 (5)
Cl1	0.0239 (5)	0.0453 (7)	0.0430 (7)	0.0024 (5)	0.0133 (5)	0.0003 (5)
N1	0.0232 (17)	0.0258 (19)	0.0262 (17)	−0.0005 (14)	0.0138 (15)	0.0010 (15)
N2	0.0294 (18)	0.0220 (19)	0.0297 (18)	−0.0025 (15)	0.0146 (16)	−0.0010 (15)
N3	0.0267 (18)	0.0282 (19)	0.0234 (17)	−0.0012 (15)	0.0126 (15)	0.0037 (15)
N4	0.035 (2)	0.0255 (19)	0.0329 (19)	−0.0025 (16)	0.0204 (17)	−0.0018 (16)
C1	0.033 (2)	0.024 (2)	0.026 (2)	−0.0014 (18)	0.0208 (19)	−0.0013 (18)
C2	0.042 (3)	0.025 (2)	0.028 (2)	−0.0020 (19)	0.021 (2)	−0.0024 (19)
C3	0.069 (4)	0.030 (3)	0.038 (3)	−0.007 (2)	0.028 (3)	0.001 (2)
C4	0.072 (4)	0.044 (3)	0.056 (3)	−0.030 (3)	0.039 (3)	−0.002 (3)
C5	0.039 (3)	0.061 (4)	0.047 (3)	−0.024 (3)	0.023 (2)	−0.005 (3)
C6	0.034 (2)	0.038 (3)	0.036 (2)	−0.005 (2)	0.022 (2)	−0.004 (2)
C7	0.048 (3)	0.034 (3)	0.040 (3)	0.012 (2)	0.019 (2)	0.011 (2)
C8	0.023 (2)	0.052 (3)	0.052 (3)	0.003 (2)	0.014 (2)	0.003 (3)
C9	0.065 (3)	0.037 (3)	0.060 (3)	−0.003 (3)	0.047 (3)	−0.011 (3)
C10	0.055 (3)	0.039 (3)	0.031 (2)	−0.012 (2)	0.017 (2)	−0.004 (2)
C11	0.046 (3)	0.030 (3)	0.038 (3)	0.005 (2)	0.023 (2)	0.006 (2)
C12	0.042 (3)	0.033 (3)	0.046 (3)	−0.012 (2)	0.022 (2)	−0.007 (2)
C13	0.039 (2)	0.029 (2)	0.020 (2)	0.0034 (19)	0.0163 (19)	0.0016 (18)
C14	0.046 (3)	0.035 (3)	0.026 (2)	0.007 (2)	0.020 (2)	0.002 (2)
C15	0.060 (3)	0.044 (3)	0.034 (3)	0.021 (3)	0.022 (3)	0.012 (2)
C16	0.097 (5)	0.031 (3)	0.054 (3)	0.008 (3)	0.052 (4)	0.011 (3)
C17	0.068 (4)	0.029 (3)	0.051 (3)	−0.003 (2)	0.043 (3)	−0.002 (2)
C18	0.048 (3)	0.032 (3)	0.035 (2)	−0.005 (2)	0.028 (2)	−0.005 (2)
C19	0.039 (3)	0.055 (3)	0.042 (3)	0.016 (2)	0.018 (2)	0.008 (3)
C20	0.043 (3)	0.050 (3)	0.057 (3)	−0.018 (2)	0.029 (3)	−0.003 (3)
C21	0.054 (3)	0.049 (3)	0.054 (3)	−0.001 (3)	0.039 (3)	−0.003 (3)
C22	0.046 (3)	0.053 (3)	0.039 (3)	−0.002 (3)	0.015 (2)	−0.010 (3)
C23	0.062 (3)	0.036 (3)	0.044 (3)	0.017 (2)	0.026 (3)	0.007 (2)
C24	0.060 (3)	0.042 (3)	0.047 (3)	−0.023 (3)	0.039 (3)	−0.015 (2)

### Geometric parameters (Å, °)

**Table:**

Ti1—N1	1.989 (3)	C10—H10A	0.9700
Ti1—N3	1.995 (3)	C10—H10B	0.9700
Ti1—N2	2.282 (4)	C10—H10C	0.9700
Ti1—N4	2.291 (4)	C11—H11A	0.9700
Ti1—Cl1	2.3374 (13)	C11—H11B	0.9700
Si1—N1	1.713 (4)	C11—H11C	0.9700
Si1—N2	1.795 (4)	C12—H12A	0.9700
Si1—C10	1.855 (5)	C12—H12B	0.9700
Si1—C9	1.861 (5)	C12—H12C	0.9700
Si2—N3	1.716 (4)	C13—C18	1.406 (6)
Si2—N4	1.789 (4)	C13—C14	1.407 (6)
Si2—C21	1.851 (5)	C14—C15	1.386 (7)
Si2—C22	1.863 (5)	C14—C19	1.511 (7)
N1—C1	1.418 (5)	C15—C16	1.383 (8)
N2—C12	1.476 (5)	C15—H15A	0.9400
N2—C11	1.479 (6)	C16—C17	1.374 (8)
N3—C13	1.419 (5)	C16—H16A	0.9400
N4—C24	1.474 (6)	C17—C18	1.380 (7)
N4—C23	1.490 (6)	C17—H17A	0.9400
C1—C2	1.403 (6)	C18—C20	1.502 (7)
C1—C6	1.406 (6)	C19—H19A	0.9700
C2—C3	1.387 (6)	C19—H19B	0.9700
C2—C7	1.500 (6)	C19—H19C	0.9700
C3—C4	1.374 (8)	C20—H20A	0.9700
C3—H3A	0.9400	C20—H20B	0.9700
C4—C5	1.367 (8)	C20—H20C	0.9700
C4—H4A	0.9400	C21—H21A	0.9700
C5—C6	1.381 (6)	C21—H21B	0.9700
C5—H5A	0.9400	C21—H21C	0.9700
C6—C8	1.501 (7)	C22—H22A	0.9700
C7—H7A	0.9700	C22—H22B	0.9700
C7—H7B	0.9700	C22—H22C	0.9700
C7—H7C	0.9700	C23—H23A	0.9700
C8—H8A	0.9700	C23—H23B	0.9700
C8—H8B	0.9700	C23—H23C	0.9700
C8—H8C	0.9700	C24—H24A	0.9700
C9—H9A	0.9700	C24—H24B	0.9700
C9—H9B	0.9700	C24—H24C	0.9700
C9—H9C	0.9700		
			
N1—Ti1—N3	135.36 (14)	H8B—C8—H8C	109.5
N1—Ti1—N2	73.69 (13)	Si1—C9—H9A	109.5
N3—Ti1—N2	101.94 (14)	Si1—C9—H9B	109.5
N1—Ti1—N4	101.77 (13)	H9A—C9—H9B	109.5
N3—Ti1—N4	74.68 (14)	Si1—C9—H9C	109.5
N2—Ti1—N4	169.84 (13)	H9A—C9—H9C	109.5
N1—Ti1—Cl1	111.41 (10)	H9B—C9—H9C	109.5
N3—Ti1—Cl1	113.23 (10)	Si1—C10—H10A	109.5
N2—Ti1—Cl1	95.13 (9)	Si1—C10—H10B	109.5
N4—Ti1—Cl1	95.00 (10)	H10A—C10—H10B	109.5
N3—Ti1—Si1	134.19 (11)	Si1—C10—H10C	109.5
N4—Ti1—Si1	138.01 (9)	H10A—C10—H10C	109.5
Cl1—Ti1—Si1	97.07 (5)	H10B—C10—H10C	109.5
N1—Ti1—Si2	130.26 (10)	N2—C11—H11A	109.5
N2—Ti1—Si2	138.34 (9)	N2—C11—H11B	109.5
Cl1—Ti1—Si2	102.91 (4)	H11A—C11—H11B	109.5
Si1—Ti1—Si2	159.95 (4)	N2—C11—H11C	109.5
N1—Si1—N2	94.26 (16)	H11A—C11—H11C	109.5
N1—Si1—C10	117.3 (2)	H11B—C11—H11C	109.5
N2—Si1—C10	108.0 (2)	N2—C12—H12A	109.5
N1—Si1—C9	114.0 (2)	N2—C12—H12B	109.5
N2—Si1—C9	114.5 (2)	H12A—C12—H12B	109.5
C10—Si1—C9	108.3 (2)	N2—C12—H12C	109.5
C10—Si1—Ti1	110.52 (18)	H12A—C12—H12C	109.5
C9—Si1—Ti1	141.20 (18)	H12B—C12—H12C	109.5
N3—Si2—N4	96.25 (16)	C18—C13—C14	119.1 (4)
N3—Si2—C21	116.0 (2)	C18—C13—N3	121.0 (4)
N4—Si2—C21	109.9 (2)	C14—C13—N3	119.9 (4)
N3—Si2—C22	114.6 (2)	C15—C14—C13	119.4 (5)
N4—Si2—C22	112.7 (2)	C15—C14—C19	118.8 (4)
C21—Si2—C22	107.2 (2)	C13—C14—C19	121.9 (4)
C21—Si2—Ti1	118.36 (18)	C16—C15—C14	121.3 (5)
C22—Si2—Ti1	134.44 (18)	C16—C15—H15A	119.3
C1—N1—Si1	124.5 (3)	C14—C15—H15A	119.3
C1—N1—Ti1	135.6 (3)	C17—C16—C15	118.9 (5)
Si1—N1—Ti1	99.43 (16)	C17—C16—H16A	120.6
C12—N2—C11	108.9 (4)	C15—C16—H16A	120.6
C12—N2—Si1	117.9 (3)	C16—C17—C18	121.9 (5)
C11—N2—Si1	112.8 (3)	C16—C17—H17A	119.1
C12—N2—Ti1	119.6 (3)	C18—C17—H17A	119.1
C11—N2—Ti1	109.2 (3)	C17—C18—C13	119.4 (5)
Si1—N2—Ti1	87.02 (14)	C17—C18—C20	119.1 (4)
C13—N3—Si2	122.5 (3)	C13—C18—C20	121.6 (4)
C13—N3—Ti1	136.5 (3)	C14—C19—H19A	109.5
Si2—N3—Ti1	99.94 (17)	C14—C19—H19B	109.5
C24—N4—C23	108.3 (4)	H19A—C19—H19B	109.5
C24—N4—Si2	113.1 (3)	C14—C19—H19C	109.5
C23—N4—Si2	117.5 (3)	H19A—C19—H19C	109.5
C24—N4—Ti1	112.7 (3)	H19B—C19—H19C	109.5
C23—N4—Ti1	116.6 (3)	C18—C20—H20A	109.5
Si2—N4—Ti1	87.61 (15)	C18—C20—H20B	109.5
C2—C1—C6	119.2 (4)	H20A—C20—H20B	109.5
C2—C1—N1	121.0 (4)	C18—C20—H20C	109.5
C6—C1—N1	119.8 (4)	H20A—C20—H20C	109.5
C3—C2—C1	119.0 (4)	H20B—C20—H20C	109.5
C3—C2—C7	119.8 (4)	Si2—C21—H21A	109.5
C1—C2—C7	121.2 (4)	Si2—C21—H21B	109.5
C4—C3—C2	121.4 (5)	H21A—C21—H21B	109.5
C4—C3—H3A	119.3	Si2—C21—H21C	109.5
C2—C3—H3A	119.3	H21A—C21—H21C	109.5
C5—C4—C3	119.5 (5)	H21B—C21—H21C	109.5
C5—C4—H4A	120.2	Si2—C22—H22A	109.5
C3—C4—H4A	120.2	Si2—C22—H22B	109.5
C4—C5—C6	121.4 (5)	H22A—C22—H22B	109.5
C4—C5—H5A	119.3	Si2—C22—H22C	109.5
C6—C5—H5A	119.3	H22A—C22—H22C	109.5
C5—C6—C1	119.4 (5)	H22B—C22—H22C	109.5
C5—C6—C8	119.5 (4)	N4—C23—H23A	109.5
C1—C6—C8	121.0 (4)	N4—C23—H23B	109.5
C2—C7—H7A	109.5	H23A—C23—H23B	109.5
C2—C7—H7B	109.5	N4—C23—H23C	109.5
H7A—C7—H7B	109.5	H23A—C23—H23C	109.5
C2—C7—H7C	109.5	H23B—C23—H23C	109.5
H7A—C7—H7C	109.5	N4—C24—H24A	109.5
H7B—C7—H7C	109.5	N4—C24—H24B	109.5
C6—C8—H8A	109.5	H24A—C24—H24B	109.5
C6—C8—H8B	109.5	N4—C24—H24C	109.5
H8A—C8—H8B	109.5	H24A—C24—H24C	109.5
C6—C8—H8C	109.5	H24B—C24—H24C	109.5
H8A—C8—H8C	109.5		
